# The Politics of Exceptionalism: Securitization and COVID-19

**DOI:** 10.1093/isagsq/ksab024

**Published:** 2021-09-22

**Authors:** Jessica Kirk, Matt McDonald

**Affiliations:** Griffith University, Australia; University of Queensland, Australia

## Abstract

The suggestion that we “are at war” with the coronavirus pandemic was not uncommon in national representations of the challenge posed by the virus. Such a representation was in turn frequently linked to the imperative of emergency responses, including expanded police powers, national lockdowns, and border closures. For theorists of securitization, this is not surprising. For them, the language of security and existential threat enables extraordinary and exceptional practices. This paper interrogates these assumptions about the performative and enabling role of securitizing language by beginning with emergency measures and asking how these were justified, how they became possible, and how prominent the language of “security” was to this politics of exceptionalism. It examines justifications for emergency responses—national lockdown and/or border closures—in the United Kingdom, Australia, and New Zealand in March 2020. Ultimately, the cases examined demonstrate significant variability in justifications for similar extreme measures. In the process, this analysis challenges core assumptions about the conditions in which extraordinary measures become possible, suggesting, in turn, the need for a context-specific understanding of both securitization and the conditions of exceptionalism.

## Abstract (fr)

L'idée que nous « sommes en guerre » contre la pandémie de coronavirus n'a pas été rare dans les représentations nationales du défi imposé par le virus. Cette représentation a à son tour fréquemment été associée à l'impératif de réponses d'urgence, notamment à l'extension des pouvoirs de la police, aux confinements nationaux et à la fermeture des frontières. Pour les théoriciens de la sécuritisation, ce n'est pas surprenant. Pour eux, le langage de la sécurité et de la menace existentielle permet des pratiques d'exception qui sortent de l'ordinaire. Cet article s'interroge sur ces suppositions concernant le rôle performatif et habilitant du langage de sécuritisation en commençant par les mesures d'urgence et en se demandant comment elles ont été justifiées, comment elles sont devenues possibles et à quel point le langage de la « sécurité » a eu son importance dans cette politique d'exceptionnalisme. Il examine les justifications des réponses d'urgence—confinement national et/ou fermetures des frontières—qui sont intervenues au Royaume-Uni, en Australie et en Nouvelle-Zélande en mars 2020. En définitive, les cas examinés présentent une variabilité considérable—dans l'articulation des et la réponse aux—justifications de mesures extrêmes similaires. Ce faisant, cette analyse remet en question les suppositions fondamentales concernant les conditions dans lesquelles des mesures extraordinaires deviennent possibles, suggérant à son tour la nécessité d'une compréhension spécifique au contexte à la fois de la sécuritisation et des conditions de l'exceptionnalisme.

## Abstract (es)

La sugerencia de que “estamos en guerra” con la pandemia del coronavirus no fue extraña en las representaciones nacionales del desafío que plantea el virus. A su vez, esta representación a menudo se relacionaba con el imperativo de las respuestas de emergencia, incluida la ampliación de los poderes policiales, los cierres nacionales y el cierre de fronteras. Para los teóricos de la titularización, esto no es sorprendente. Para ellos, el lenguaje de la seguridad y la amenaza existencial da lugar a prácticas extraordinarias y excepcionales. Este artículo analiza estos supuestos sobre el papel performativo y habilitador del lenguaje de la segurización, comenzando por las medidas de emergencia y preguntando cómo se justificaron, cómo se hicieron posibles y qué importancia tuvo el lenguaje de la “seguridad” en esta política de excepcionalidad. Se examinan las justificaciones de las respuestas de emergencia, cierre nacional y/o cierre de fronteras, en el Reino Unido, Australia y Nueva Zelanda en marzo de 2020. En última instancia, los casos examinados demuestran una importante variabilidad, tanto en la articulación como en la respuesta a las justificaciones de medidas extremas similares. En el proceso, este análisis pone en tela de juicio los supuestos básicos sobre las condiciones en las que se hacen posibles las medidas extraordinarias, y a su vez sugiere la necesidad de una comprensión específica del contexto de la titularización y de las condiciones de la excepcionalidad.

Theorists of securitization argue that the language of security and threat serves to enable extraordinary measures to protect or advance security. The suggestion here is that once an existential threat is presented by political elites and accepted by a relevant audience, policymakers and practitioners are able to pursue measures (to protect security) that otherwise may not have been countenanced. The COVID-19 crisis provides a unique opportunity to reflect upon and interrogate this assumption. Extraordinary measures—restrictions on movement and border closures in particular—have been enacted in a range of states, with some analysts suggesting that narratives of “war” were particularly prominent in justifying these measures. While most analyses of securitization processes focus on the language employed and follow these through to subsequent measures,^1^ this paper works backward: we begin with the enactment of extraordinary measures and then turn our attention to the language and justifications that preceded these, exploring the prominence of representations of security and threat. Such an approach is possible given the similarity of measures undertaken but also their scale and significance, necessitating some form of public justification.

This paper therefore examines justifications for extraordinary measures in response to the COVID-19 pandemic, with a focus on the role of representations of security or threat in enabling such responses. In attempting to reflect more broadly on the politics of exceptionalism as envisaged by theorists of securitization, we examine justifications for extraordinary measures in three different countries: the United Kingdom, Australia, and New Zealand. All three pursued extraordinary responses in the form of national lockdowns and/or border closures, particularly in mid-late March 2020, albeit with different sets of results and at different stages of the pandemic. Similarities in political system and language also enable meaningful comparison in these instances. More specifically, we explore statements outlining and justifying the most significant extreme measures undertaken by each state—national lockdown in the United Kingdom (March 23); border closures (March 20) and lockdown (March 23) in Australia; and border closures (March 19) and national lockdown (March 25) in New Zealand. The period of analysis across the three states is a two-week period from March 16 to March 30, 2020. We focus on statements from the Prime Minister in each country in outlining and justifying measures, and are particularly attentive to the use of the terms “security” and “threat.”^2^ In the process, however, we also reflect on the overarching thrust of justificatory attempts: how, in broad terms, were emergency measures sold?

The paper proceeds in three parts. In the first, we contextualize our exploration of the securitization of COVID-19 in these cases by outlining existing engagement with the relationship between security and disease, in both the academy and practice. The second section further builds the foundation for analysis by introducing the securitization framework, with a particular focus on its assumptions and claims regarding the relationship between securitization and exceptionalism. Together, these sections provide a necessary contextual basis for the third section—the focus of our analysis. In this section, we examine key statements from political leaders in the United Kingdom, Australia, and New Zealand that introduced, outlined, and ultimately justified extraordinary measures such as national lockdown and the closure of borders. We conclude by reflecting on the implications of this analysis for the way we think about the performative effects of security representations and the conditions in which exceptional practices become possible.

In critically engaging assumptions about security language and exceptionalism, this paper aims to contribute both to our empirical understanding of different (justifications for) exceptional measures in the face of the COVID-19 pandemic and to theoretical debates about how “securitizing” language operates and how exceptionalism is enacted and justified. While those developing and applying securitization theory frequently work with the assumption that securitization entails elites justifying emergency measures through depictions of existential threat, the empirical analysis to follow ultimately questions this assumption. The UK example in particular challenges the idea that elite actors employ the language of security and threat to enable measures that might not otherwise have been countenanced, while the New Zealand case suggests that extraordinary measures may be possible without the facilitating language of security. In the process, this analysis points to the importance of context-based analysis of the politics of exceptionalism and the politics of security.

## Disease and Security

Disease has long been considered a high-level issue of state concern, even security. Recent decades, however, have seen a significant rise in the attention paid—by both scholars and practitioners—to the explicit nexus between disease and security. Part of this can be linked to a confluence of global health crises during this period: HIV/AIDS in the 1980s onward, the threat of bioterrorism highlighted by the Anthrax attacks of 2001, SARS in 2005, H1N1 in 2009, Ebola in 2014, Zika in 2016, and today, COVID-19. Each event catalyzed public awareness of disease's potential—and global—threat and the urgency of adequate preparedness and response, provoking the formation and alteration of policies and the shift of health into “high level” agendas. Yet another, equally crucial component of this lies in the expansion of security. A series of critical interventions and new concepts in the 1990s challenged the conceptual confinement of security to the “threat, use, and control of military force” (Walt 1991, 212), and presented an understanding of security as encompassing a far broader range of issues, referent objects, and practices. Disease entered this field via two pathways: the individual-centred emphasis on its threat to human security (see Chen, Leaning, and Narasimhan 2003; Ogata and Sen 2003) and via calls for greater attention to be paid to the threat of “emerging and re-emerging infectious diseases” to “the political, economic, and strategic interests of the state” (Dupont 2001, 6; see also Lederberg, Shope, and Oaks 1992; Garrett 1996; Price-Smith 2002). While both understandings of disease as a security threat persist (see Davies 2010; McInnes 2014), it is the latter—in combination with the aforementioned crises—that has ultimately taken precedence amongst states, organizations, and scholars (Rushton 2011; Weir 2014).

This approach views (particular) diseases as threats to national security. Advocates emphasize the potential of disease outbreaks to “hollow out” military capability (Singer 2002, 145–46; see also Petersen 2002, 75–78), including its threat to troops should they deploy into an outbreak area (Dupont 2001; Elbe 2002). They also point to threats to both economic and political stability associated with disease, with the potential for disruption in the “just-in-time” economy (Osterholm 2007, 55) and even the possibility of state collapse (Ostergard 2002; Price-Smith 2009, 89–114). Bioterrorism is another common linkage, with governments in particular linking naturally occurring diseases to security through the risk of non-state actors “weaponising” a spreading infection (USNSC 2009, i). However, the veracity of these linkages is contested. Militaries are not as dramatically undermined by disease as suggested (Barnett and Prins 2006; McInnes 2006; McInnes and Lee 2012, 149–55). Disease outbreaks are yet to *cause* state collapse, though they do facilitate significant internal instability (Price-Smith 2009, 3–4, 115–16, 212). And bioterrorism, particularly that by non-state actors, is a far more complex endeavor than assumed (Koblentz 2009, 2014). In addition, the diseases typically presented and addressed as security concerns are not always those with a high mortality or morbidity rate, nor those that necessarily present a significant danger to the fearful state. Ultimately, much of the public rhetoric surrounding disease's threat to national security tends to focus less on concrete connections and more on the fears and values of the societies facing them (Alcabes 2009, 4; see also Nunes 2017, 6–7; Wald 2008). Accordingly, while the disease–security nexus and its emphasis on national security remain important, there appears to be a crucially subjective component to this.

For many scholars of global health security, securitization theory offers the best lens to explore this. Securitization shifts the focus from trying to ascertain the objective “securityness” of disease to exploring how particular diseases are *constructed* as security threats. The linkages between disease and national security thus do not necessarily need to be valid. Instead, their “securityness” is established when an actor in a position of political authority (whether formal or informal) “speaks security” and a relevant “audience” accepts this connection, tolerating “extraordinary measures that otherwise would not have been successful” (Wæver 2011, 469). Scholars exploring this securitization of disease have thus focused on how elites have represented particular diseases as security threats, exploring the construction of HIV/AIDS (Sjöstedt 2008; Herington 2010; Lo 2015), SARS (Wishnick 2010), H5N1 influenza (Elbe 2010; Herington 2010; Hameiri and Jones 2015), H1N1 influenza (Abraham 2011), Zika (Ventura 2016; Wenham and Farias 2019), Ebola (Honigsbaum 2017; Enemark 2017a), and now COVID-19. Some have even taken a wider view, arguing that disease itself has been securitized, whether through a snowballing effect of various securitizing moves (Fidler 2003; Kamradt-Scott and McInnes 2012) or through the concerted efforts of Western elites to privilege their concerns (Davies 2008; Weir 2014).

Key conclusions arise from these analyses. First, the types of diseases constructed as security threats tend to be those which invoke a level of atavistic dread, or conform to what Priscilla Wald (2008, 2) has termed the “outbreak narrative”: “an evolving story of disease emergence” with a “formulaic plot” of emerging infection, global spread, and eventual containment via “the triumph of science and epidemiology.” Second, despite the potential for securitization to draw neglected diseases or sufferers into the realm of “high politics,” global health security agendas remain firmly anchored to the plight faced by *Western* states from “dreaded diseases.” Enemark (2017b, 139) terms this the “attention or neglect” dilemma, where the focus on outbreaks diminishes the attention to more long-term issues. Nunes (2016, 550) locates this in securitization itself: it is “markedly solipsist” and concerned fundamentally with the protection of the “Self” from the “Other.” Finally, outbreak responses based on securitization tend to prioritize technological “quick fix” solutions like pharmaceutical development (Elbe 2010, 2014; Roemer-Mahler and Elbe 2016, 491–94; Enemark 2018), denial of civil liberties such as mandatory quarantines and policing of behavior (Enemark 2009, 199–205; Nunes 2014, 64–66; Hills 2016), extensive biopolitical interventions on the population (Elbe 2005, 2009; Enemark 2017b), and “militarised” interventions under the claim of “emergency” (Benton 2017, 39–44). This emphasis shares a foundational assumption with securitization theory: security politics enact the politics of exceptionalism.

## Securitization and Exceptionalism

Securitization theory, in its most basic form, considers security as intersubjectively constructed via speech acts. A securitizing actor—typically a member of the political elite keen to enable particular forms of response—presents an issue as a threat to security, either explicitly or implicitly through presenting an issue through “the logic of war—of challenge-resistance (defence)-escalation-recognition/defeat” (Wæver 1995, 54). This, in turn, significantly elevates an issue's political significance, ascribes a particular meaning to it, and suggests particular, “extraordinary” responses. Success in this, however, is claimed to lie within its acceptance by a relevant audience, which permits “the breaking of rules,” or emergency politics, as a legitimate response (Buzan, Wæver, and de Wilde 1998, 25). The emphasis of the framework—and the empirical research applying it—is thus typically on this language and process of “speaking” security and its apparent “success” with a relevant audience, though the significance of the latter tends to differ depending on the theorist.

However, while this suggests an approach to security's construction broadly consistent with constructivist thought, the conceptualization of security itself (what precisely is implemented) in securitization theory is far more in line with that of political realism. For the founding Copenhagen School, security is not wholly malleable or devoid of meaning. Instead, it is bound by “a set of connotations that it cannot escape,” specifically related to national security (Wæver 1995, 47, 52–53). As such, it invokes an unparalleled sense of urgency that allows securitizing actors a similarly unparalleled right to act as “[i]f we do not tackle this problem, everything else will be irrelevant (because we will not be here or will not be free to deal with it in our own way)” (Buzan, Wæver, and de Wilde 1998, 24). As Michael C. Williams (2003) and Jef Huysmans (1998) have argued, this aligns with the work of Carl Schmitt on exceptionalism. Exceptionalism, in brief, refers to the reproduction of the friend/enemy antagonism Schmitt (2010 [1932], 29) saw as fundamental to politics, intensifying it to its most extreme point of violence and war and using this to justify the decisionist power of the sovereign. Decisionism, as defined by Huysmans (1998, 582), is “the moment of political creation *ex nihilo*.” The old or everyday rules are observed (by the sovereign) as inadequate in the face of the existential threat, and new rules, new truths, and new decisions not bound by the old must be enacted. In Schmitt's (2005 [1922], 7) words, the sovereign “decides whether there is to be an extreme emergency as well as what must be done to eliminate it.” The Copenhagen School, in translating this into securitization, considers this as what “security” does. Security provokes the possibility of violent death, relying on the “mass identity” of the unit and “self-enforcing rivalries with other limited collectivities” as integral for success. This threat of immediate and violent death at the hands of an “enemy” “lift[s an issue] above politics,” essentially producing a separate, exceptional space in which political elites can act unfettered (yet legitimately) by “normal politics” (Buzan, Wæver, and de Wilde 1998, 26). It is thus a moment of sovereign power: political elites “decide” the threat (through their securitizing moves) and the appropriate response. Ultimately, securitization theory is not only the intersubjective construction of security, but the construction of exceptionalism. While possible that exceptional practices are encouraged or even compelled by other actors, the framework remains predominantly focused on sovereign decisionism, leading many applications—particularly those examining outbreak responses—to focus on elite attempts to justify or enable emergency measures (see McDonald 2008). This also provides a rationale for our focus on elite attempts to justify preferred policy responses in the context of the COVID-19 pandemic.

In light of the above, it is little surprise that many securitization scholars are deeply cynical about, if not entirely hostile to, the enactment of security politics. Indeed, advocates of securitization theory view the construction of security as a negative, with an “abstract preference” for desecuritization (Wæver 2011, 469). This is, of course, not universal, with some highlighting the potential benefits of securitization (Elbe 2006) and others noting that securitization may even be morally obligatory in the face of extreme threats (Floyd 2019). Yet most point to exceptionalism through securitization as a troubling and unwanted development. Three concerns are common here. First, scholars highlight the potential for “normal” liberal politics to be overturned in the name of “emergency.” The denial of civil liberties is a frequent concern, with concrete examples including the restriction of movement, arbitrary detention and interrogation, limitations to the freedom of speech, invasive surveillance, the use of force (including the military) to ensure compliance, and the relegation of some to a position of “bare life” (Agamben 1998, 2005; Huysmans 2004; Van Munster 2004; Dean 2007). Some also note the potential for these emergency measures to continue beyond the immediate emergency. Scholars examining the “War on Terror,” for instance, have highlighted the pervasive nature of surveillance and border controls (Van Munster 2004; Aradau and Van Munster 2008; Neal 2009).

A second but related issue is the expansion of unrestrained sovereign power legitimized by securitization. Noteworthy considerations here include the tendency to bypass processes of democratic deliberation and diminished transparency in decision-making (Huysmans 2004, 327–36). The third and final worry related to securitization's enactment of the exception is the propensity for exclusionary politics grounded in the friend/enemy antagonism. As Sara Davies (2010, 18–19) laments, in global health this results in a tendency to treat disease as “a foreign enemy to be vanquished or contained.” Joao Nunes (2016, 551) takes this a step further, highlighting the securitized narrative as one that presents those suffering from disease as the threat to be watched and contained. In essence, there is a direct connection made between the claim to security and the enactment of exclusionary, illiberal, and decisionist exceptionalism, wherein the process of constructing an issue as one of security is assumed to lead to the potentially dangerous politics of emergency.

The nascent work on the securitization of COVID-19 is no different in its concerns with exceptionalist power. Some have focused on the more overt cases of this, exploring the use of elite securitizing language to justify moves toward autocratic governance, with Turkey and the Balkans being popular examples for this kind of analysis (Molnár, Takács, and Harnos 2020; Vankovska 2020). A few have emphasized the way securitization has expanded police enforcement powers (Parker, MacGregor, and Akello 2020; Stott, West, and Harrison 2020), while Hannah van Kolfschooten and Anniek de Ruijter (2020) have similarly offered analysis of the expansion of surveillance powers. Others have explored the forms of exceptionalism unique to COVID-19. Joao Nunes (2020, 1–2), for instance, argues that not only has COVID-19 been labeled a security threat, but we have also observed the “securitization of the circulation of persons and social contact,” a representation of modes of being as dangerous. The resulting “states of exception” are thus those of denying these “normal” ways of being, which have significant social and economic consequences, as well as opening spaces for a new economic truth to be formed. Murphy, M (2020) offers a clear example of this in education, arguing that face-to-face schooling is one of these modes of being that has been securitized alongside COVID-19. The most prominent focus here, however, has been on bordering practices. Triandafyllidou (2020, 261) argues that COVID-19’s securitization has strengthened exclusionary understandings of belonging, formalized in the “unprecedented decisions” to close borders to noncitizens—a feature of responses in two of the three states analyzed here. Others have expanded on this line of argument, but stress that COVID-19 also highlights the multiplicity of bordering practices under securitization, including those of rescuing citizens, limiting exports of vital (medical) goods, and the expansion of “geofencing” (Ferhani and Rushton 2020; Liu and Bennett 2020).

Perhaps a more common area of focus here—particularly in public commentary—is the use of “war metaphors” to describe COVID-19’s threat and the apparent consequences of this. As many have pointed out, political elites have frequently described their responses to the pandemic as akin to fighting a war (Caso 2020; Haddad 2020; Heffernan 2020; Hoffman Pfrimer and Barbosa 2020; Musu 2020; Serhan 2020; Tisdall 2020; Wilkinson 2020). While some suggest that this may have potential benefit in communicating the gravity of the crisis (Serhan 2020), most view it as a troubling green light for the enactment of exceptionalism. Musu (2020) points to it as leading to the expansion of powers to “detain and isolate people, ban public gatherings including protests and shut down ports and airports.” Tisdall (2020) highlights its use as justification for the creation of new, opaque emergency powers and postponing elections. Hoffman Pfrimer and Barbosa (2020, 138–39) argue that it enables the involvement of military officials (and their militarized responses) in the public health response in Brazil, with Heffernan (2020) suggesting that the war metaphor engenders a broader logic of “command-and-control” over civilian populations. And many have highlighted its role in creating “enemies” of those either suffering from the disease or vaguely connected to the pandemic's original source. Eric van Rythoven (2020), for instance, notes a spike in hate crimes against Asian Americans as evidence of “what's wrong with the war metaphor,” and Elena Sondermann and Cornelia Ulbert (2020) use it as a marker of why this “thinking in threats” is a threat in of itself.

While this work has provided important insights into the politics of COVID-19, it is also premised on an assumption of the direct causal linkage between the elite invocation of security and extreme exceptionalist measures. Grounding these claims is a suggestion that the extremes of exceptionalism inevitably follow—and are dependent upon—the claim to security, especially when enacted through the war metaphor. Moreover, this is overwhelmingly presented as a negative. While there is often a brief recognition that the unprecedented nature of the COVID-19 pandemic requires an equally strong response, commentary has largely taken the illiberal and decisionist potential of exceptionalism as the inevitable end project. However, neither of these claims is necessarily proven. The securitization literature is characterized by contestation over the precise effects of securitizing language, particularly in terms of its direct production of emergency measures (McDonald 2008; Vuori 2017). A substantive body of work within this field has highlighted the need to add considerations of context, audience, and even other securitizing actors to adequately capture this process (Bigo 2002; Balzacq 2005; McDonald 2008; Trombetta 2008; Ciuta 2009). And the literature around exceptionalism has revealed that the decisionist and illiberal extreme identified by Schmitt is not the only form it can take, with more nuanced forms *between* “normality” and “exception”—such as risk and its management—evident (Abrahamsen 2005, 59; Johns 2005; Neocleous 2006; Doty 2007, 2009; Neal 2009; Kirk 2020). Indeed, some have even presented competing understandings of security that take the base idea of exceptionalism and suggest more beneficial manifestations (Doty 1998; McDonald 2003; Williams 2015). This all suggests a compelling rationale to systematically examine the relationship between the representation of COVID-19 as a security threat on one hand and the emergency measures that have been enacted on the other.

## Justifying Exceptionalism: Comparative Analysis

As noted at the outset, this paper examines the three cases of the United Kingdom, Australia, and New Zealand, with a particular focus on how emergency measures associated with lockdown and border closures were justified by political leaders (in March 2020).

Of course, national-level comparisons will always be imperfect. All three countries discussed here are constitutional monarchies with Westminster political systems, yet forms of governance and jurisdiction in each differ in important ways. While responsibility for managing lockdown in New Zealand was centralized, for example, this was largely the jurisdiction of state governments in Australia. State governments in the case of Australia also have responsibility for health, including hospital management. All countries discussed here are relatively wealthy states with similar levels of economic development, yet have different capacity (and again jurisdiction) when it comes to emergency health and hospital beds, for example. All three countries are island states, implying increased capacity to manage and close off borders to foreign entry. But of course in practice, and despite a similar geographic size, everyday population movement to/from the United Kingdom and New Zealand differs vastly, linked to population size, proximity to other countries, and their respective role as countries of transit. And in the particular case of COVID-19, timing was significant. While emergency measures were enacted at broadly similar times (March 2020), this similarity masked significant differences in the pandemic's trajectory in all three states—in the United Kingdom, national lockdown was predominantly responsive and followed a significant increase in case numbers. In New Zealand, by contrast lockdown was predominantly preventative, with only 102 cases (and no fatalities) recorded at the point lockdown was announced (McKay, Ben 2020). New Zealand's experience also arguably involved greater capacity to learn from policy responses in countries that had been affected by the virus earlier, and a public arguably more willing to countenance extreme measures given the effects of the pandemic evident in other states. Australia, meanwhile, sat in between these responses: less reactive than the United Kingdom in pursuing emergency measures—occurring before large numbers of cases and fatalities—but less preventative than New Zealand, responding instead to projections of exponential case growth.

These important points of distinction notwithstanding, the cases discussed here do involve emergency responses, and even similar (if not identical) forms of emergency responses—border closures and lockdowns. To this end, a comparison of these states provides a unique basis for interrogating how similar sets of measures were justified across the three states, and what role claims of security or threat played in enabling emergency measures.

### United Kingdom: Exceptionalism and Securitization (Eventually)

In comparative terms, the United Kingdom has been among the hardest hit countries in the world by the coronavirus. By early 2021, in the midst of a third wave of a virus, the United Kingdom was recording over 50,000 new cases and over 1,000 fatalities per day (Sky 2021). The cumulative total of cases to that point was nearing 3 million, with over 77,000 deaths. And of course the Prime Minister himself—Boris Johnson—figured among those statistics after being hospitalized with coronavirus in early April 2020. In this sense, the United Kingdom is something of an “outlier” in comparison to the other countries analyzed here. While neither Australia nor New Zealand were among the top 100 states in the world in terms of total cases, the United Kingdom was fifth in the world for total cases at the beginning of 2021, with higher rates of infection as a percentage of its population than all other countries in that top five with the exception of the United States.

Despite this, the United Kingdom first initiated what might be described as exceptional practices—expansion of government powers to restrict movement and then national lockdown—at a similar time to the other countries addressed (mid-late March 2020). Indeed more than either of the other states addressed here, significant pressure was building on the United Kingdom to both pursue extraordinary measures and in particular to reject suggestions that the United Kingdom might consider business-as-usual strategies with the ultimate goal of achieving “herd immunity.” As one analyst noted in mid-March 2020 (McMahon 2020), the “(British) government has been slow to implement the same kind of tough social distancing measures its European neighbours, including Italy, Spain and France, have adopted, such as a ban on mass gatherings and shutting down restaurants, museums and all non-essential shops.” Meanwhile 200 scientists in the United Kingdom, in response to the United Kingdom’s Chief Scientific Officer suggesting that “herd immunity” might be an appropriate strategy of response to the COVID-19 outbreak, penned an open letter condemning the strategy and arguing that “additional and more restrictive measures should be taken immediately” (in McMahon 2020).

This might suggest—certainly in contrast to other countries—that the British government needed to do less to convince the broader population of the imperative of such measures. Indeed at the point that Health Minister Matt Hancock outlined the initial Coronavirus Emergency Bill introducing restrictive measures in UK Parliament—on March 16, 2020—over 1,500 people had been infected and more than 50 had died as a result of the illness. By the time Prime Minister Boris Johnson announced lockdown in a national address a week later, over 300 people had died as a result of the illness (Dickson 2020). These two speeches, outlining and justifying extraordinary measures in response to the COVID-19 outbreak, are the focus of analysis here.

When introducing the Coronavirus Bill, which gave government agencies the right to detain people, ban public gatherings, and shut down airports and ports, Health Secretary Matt Hancock began a relatively short speech by indicating that the goal of government policy in response to the pandemic was “to protect life.” He acknowledged here that “the measures I have outlined are unprecedented in peacetime,” before noting that “we will fight this virus with everything we have. We are in a war against an invisible killer and we have to do everything we can to stop it” (Hancock 2020). Clearly, representations of security and threat were central to the case made for restrictive measures.

In his statement outlining the lockdown on March 23, meanwhile, the Prime Minister commenced his address by noting “the coronavirus is the biggest threat this country has faced for decades” (Johnson 2020). The measures he outlined included closure of shops, limits of movement to specific contexts—one form of exercise per day, access to medical services, travel to and from work—and restrictions on the number of people at any gathering. The Prime Minister called on the people of Britain to work together to respond to the “national emergency,” invoking military terminology in suggesting that “. . . in this fight we can be in no doubt that each and every one of us is directly enlisted” (Johnson 2020).

In both of the above cases, representations of threat and even references to experiences in war were a feature of attempts to outline and justify the extraordinary measures undertaken. The Health Minister and Prime Minister variously invoked war, employed war metaphors, and emphasized the threat posed by the pandemic in justifying the measures the United Kingdom was planning to pursue (see Freedman 2020; Musu 2020). But did this have the effect of enabling emergency measures that otherwise might not have been countenanced, as securitization scholars might suggest? Such an argument is difficult to sustain in Britain's case given the pressure already being placed on the UK leadership to escalate the national response, noted above. In this case, it may be that language *followed* public pressure for extraordinary measures, rather than language *enabling* support. Indeed, it seems here more plausible to argue that the use of this language reflected a concern with redressing perceptions that the government was taking the issue less seriously than it should be. It is telling to note that one day prior to the announcement of the Coronavirus Emergency Bill, a national poll indicated that only 44 percent of Britons felt the government was handling the coronavirus outbreak well, while less than 40 percent of Britons indicated that they trusted the advice given to them by either the Prime Minister (36 percent) or the Health Secretary (37 percent) (Helm 2020).

Despite the language employed by the Health Minister and the Prime Minister, and the restrictions put in place, the majority (51 percent) of respondents in a national opinion poll conducted during the lockdown suggested that the United Kingdom was not firm enough in responding to the coronavirus (Coates 2020). In this context, again the role of (elite) securitizing moves in enabling emergency measures appears questionable—if anything, the British population seemed more likely to call for extraordinary measures than the Government was to pursue them.

As noted, the United Kingdom has been particularly affected by the coronavirus outbreak in comparative terms. Aside from higher numbers and fatalities over time, the United Kingdom has experienced more waves and the reimposition of national-level restrictions than the other countries examined here—Australia and New Zealand. And it should be noted that the United Kingdom is the only country of the three analyzed here that has not initiated border closures at the national level. These all suggest a government apparently less concerned with addressing the immediate challenge of COVID-19 than other states, even one lagging behind national expectations for emergency action. Rather than securitization driven by the political elite enabling emergency responses then, the UK case suggests a government using the language of security, threat, and war to communicate urgency consistent with societal expectations. In the process, this case questions the role of elite securitizing language in rendering extraordinary measures possible, suggesting even that such language might be incidental to (pre-existing) support for such measures.

### Australia: Partial Securitization?

In contrast to the United Kingdom’s delayed and reactive response and its tragic consequences, Australia's COVID-19 response has widely—at least up to the point of the vaccination program—been lauded as timely and successful (Child et al. 2020; Holden 2020; Patrick 2020), resulting in an overall number of cases lower than daily increases seen in the United Kingdom.^3^ Australia started with selected, but rolling border closures from February 1, with complete border closures to nonresidents announced on March 19. The government implemented a national lockdown, with closures and movement restrictions announced and implemented on March 22 and 23. Notably, Australia also developed a new institutional mechanism to facilitate coordinated action: the National Cabinet.

The National Cabinet included the Prime Minister, Federal Health Minister, and the Premiers/Chief Ministers of each state and territory. In Australia, responsibility for health systems and measures such as quarantine, border controls, and lockdowns belongs to states and territories rather than the federal government, a division of power that was only reiterated in the major COVID-19 response plan (DOH 2020, 16–18). Yet while some state leaders used this power to tighten restrictions or enact additional ones, the major early decisions regarding border closures and national lockdowns (those relevant to this analysis) were made through collaboration within the Cabinet and announced by the Prime Minister. Indeed, during this period, it was argued that COVID-19 represented an “apolitical” crisis, one that demanded unification in the name of emergency (March 15). This unification behind the federal government, and the Prime Minister as the key actor, aligns with securitization theory's emphasis on *decisional* power (Buzan, Wæver, and de Wilde 1998).

In announcing these exceptional measures, Prime Minister Morrison followed the UK emphasis on the need to protect and save lives, with the terminology of war increasingly used to depict the danger. While the word “threat” was not often used (one notable use was March 18, however, when the Biosecurity Act was invoked to close borders; see Morrison 2020b), Morrison referred to the “battle” Australia faced against the virus, especially in his important speeches on lockdown measures (Morrison 2020c, 2020d), even referring to Australians as “enlisted” in this battle (March 22) and the measures as “weapons” against the virus (2020c). March 23 even saw an explicit comparison, with the “test” of COVID-19 observed as reminiscent to that of World War II, and powerful cultural discourses surrounding Australia's wartime history were invoked. In asking for compliance and understanding, Morrison asked Australians to “summon the spirit of the Anzacs, of our Great Depression generation, of those who built the Snowy, of those who won the great peace of the Second World War and defended Australia” (Morrison 2020d).

However, while Australia shared the war terminology with the United Kingdom, there were also crucial differences in this framing. Rather than presenting COVID-19 as an evident and imminent threat, Morrison's focus was on the protection of select parts of the population—the “most vulnerable” and “the most at risk”(Morrison 2020a)—from potential danger. The terminology of war was thus merged with that of risk. Representations of solidarity—while including those of war legacies and national unity behind the executive—also involved calls for “care, compassion, and respect” (Morrison 2020c) and citizen responsibility to “follow[. . .] common sense rules and do[..] the right thing” (Morrison 2020a). In the process, the government recognized the economic cost of the closures and lockdowns. Indeed, Morrison depicted this as another front in this battle, with some Australians “in the frontline of the blows that we will experience, the economic blows” (Morrison 2020c). The Morrison government recognized that a form of exceptionalism—with all its benefits *and* dangers—was being implemented through these policies, and the Prime Minister made concerted efforts to represent this as a necessary “sacrifice” to protect “our” vulnerable from potential harm, akin to that made by the Anzacs (Morrison 2020c).

The measures undertaken enjoyed broad public support in Australia. One national poll showed Australians overwhelmingly in favor of the restrictions being enacted (Murphy, K 2020b) and growing approval for the government's response during the lockdown (Farr 2020). Crucially, these results also showed that Australians were increasingly anxious about the pandemic during this same period, with decreasing numbers believing the government's response was an overreaction (18 percent on March 30, down from 33 percent) and increases in those describing themselves as “very concerned” (53 percent on March 30, up 14 points from the previous week) about the virus (Murphy, K 2020a, 2020b). This, of course, cannot be attributed directly to the speeches and conferences of the Prime Minister, but indicates a growing sense of danger and an acceptance of the need for emergency action.

Australia's response thus initially appears in line with the securitization framework: emergency measures were outlined and justified through representations of war and danger, seemingly convincing the population of their necessity even in the context of economic cost. Unlike the United Kingdom, these actions—while not as early or significant as some would have liked—were enacted prior to large increases in cases or fatalities and widespread public demand.

Yet this also involved crucial divergences from the securitization framework. Representations of war were premised not on the idea of COVID-19 as an imminent threat, but as a risk to vulnerable members of the community. The use of executive power (and the centralizing of said power) existed side by side with calls for citizen responsibility and solidarity. It could also be argued that the Australian government demonstrated a level of concern with the *kind* of exception being enacted. More businesses were permitted to continue operating than in New Zealand (see Fuller 2020), for instance, and the government—through the National Cabinet—insisted on schools remaining open, arguing that the cost of closures to children, businesses, and workers was unjustifiable (March 15). Moreover, like the United Kingdom, the role of the political elite in enabling exceptionalism remains questionable. Polls indicated that less than half of Australians (45 percent) believed the government's response was “good” or “very good” prior to the March 23 lockdown (Murphy, K 2020a). The government also saw significant public backlash over perceived complacency in early decisions and incidents, such as the Prime Minister seeking to attend a rugby league match just two days before such mass gatherings would be banned (McPhee 2020). These imply that there was a level of reactivity to the public's perception of the danger. Like in the United Kingdom, this challenges the idea that exceptionalism *followed* the government's securitizing language. Ultimately, the Australia case does not fit neatly into the Copenhagen School framework.

### New Zealand: Exceptionalism without Securitization

On March 14, 2020, New Zealand Prime Minister Jacinda Ardern announced border restrictions in response to the coronavirus pandemic that were among the toughest in the world. She indicated that anyone arriving in the country would need to self-isolate for two weeks. At the time, New Zealand had only six coronavirus cases. Within a week, with active cases up to twenty-eight, the borders had been closed to all nonresidents. By the time the Prime Minister announced that New Zealand would be entering its most significant stage of lockdown—Stage 4—on March 23, New Zealand still had just 102 cases (see Hollingsworth 2020).^4^ It was a set of measures, in the words of the Prime Minister, that represented New Zealand's commitment to “going hard, and going early” (Ardern 2020a).

New Zealand's response to COVID-19 has been lauded as one of the most successful in the world. This is certainly the case when assessed against case numbers, fatalities, and even pressure on health systems. By the end of 2020, New Zealand (with a population of 5 million) had recorded less than 2,200 cases and twenty-five fatalities, the latter at a rate of five deaths per million of the population. And New Zealand did not experience the genuine pressure (even crisis) on hospitals and emergency health capacity experienced in some of the worst-hit states, including the United Kingdom. Of course, these measures involved economic costs. A Reserve Bank assessment indicated that Stage 4 restrictions of lockdown—measures in place for 5 weeks from March 25, 2020—risked resulting in a 37 percent reduction in gross domestic product (GDP) (Stannard, Steven, and McDonald 2020). The economy did indeed slip into its deepest recession since 1987 in June 2020, with the economy shrinking by 12.2 percent between April and June (BBC 2020). And while the end to lockdown measures eased these effects nationally, ongoing border closures have significantly impacted New Zealand's tourism sector—its largest export market (Hollingsworth 2020).

Given these effects and their implications—especially in the context of what was at the time of their announcement and implementation a comparatively minimal number of cases-— how can we make sense of the means through which such measures were rendered possible? Can we see evidence here of securitization?

Even a cursory analysis of the Prime Minister's statements regarding the implementation of border control and lockdown measures suggests a decidedly marginal role for direct representations of security or threat. This was particularly evident in three key speeches examined here: the announcement of border closures to all nonresidents (March 19), the outline of proposed lockdown measures (March 21), and confirmation of the implementation of those lockdown measures (March 23).

Rather than emphasize the *threat* posed by COVID-19, Prime Minister Ardern reiterated her concern with managing (even preventing) the *risk* of coronavirus spread through fast and significant early intervention. From the perspective of securitization theory, this is not an insignificant distinction. As Jessica Kirk (2020), Olaf Corry (2012), and Andrew Neal (2009)—among others—have argued, conceptualizations of risk often suggest different forms of response more closely aligned to regulation than extraordinary measures. Despite this, and illustrating the different performative or enabling effects of representations more broadly, Prime Minister Ardern noted that “at no time in New Zealand's history has a power like this been used” (Ardern March 19, 2020).

The Prime Minister noted the “risk” associated with the importation and spread of coronavirus—linked to international arrivals—on several occasions in the announcement of border closures. She identified the “very serious risk” the coronavirus pandemic posed, the “unacceptable risk” of travelers bringing in the virus, and the “need to continue to make further decisions and further restrictions to limit the risk of people bringing the virus into New Zealand” (Ardern March 19, 2020). Management of risk rather than representation of imminent threat was therefore central. Crucially, the term “threat” appeared to be studiously avoided in statements associated with outlining emergency measures, at least in comparison to statements from UK Prime Minister Boris Johnson. While Johnson's address to the nation to announce lockdown measures on March 24 began by noting that “the coronavirus is the biggest threat this country has faced for decades” (Johnson 2020), Ardern's core statements outlining the proposed measures (March 21) and confirming their implementation (March 23) contained no use of the term “threat.” In the aftermath of Stage 4 lockdown measures, the Prime Minister did note that “we will do all we can to ensure we fight the economic impacts of the virus in the same way we did the health threat” (in Hollingsworth 2020). Yet this was—as noted—after the extraordinary measures to address COVID-19 had been introduced. And the means envisaged to address these economic impacts defined agency and means in terms of citizen participation (rather than executive power) and solidarity— “with unity, with fast support, by looking after each other” (in Hollingsworth 2020).

In broader terms, the rationale for the interventions outlined by the Prime Minister—both border closures and lockdown—was saving lives. Ardern noted that “if community transmission takes off in New Zealand the number of cases will double every five days. If that happens unchecked, our health system will be inundated, and tens of thousands of New Zealanders will die” (Ardern 2020b). The case made by the Prime Minister was that rapid and significant action, extraordinary in historical terms, was necessary to protect the lives of the most vulnerable members of society. New Zealanders were asked not to panic (in RNZ 2020), to act in solidarity, to “be kind,” and to “look after one another” (Ardern 2020a).

Strict lockdowns and border closures were enacted in New Zealand at a point in the transmission and progress of the virus well before that of other states. These were also outlined and pursued well before (widespread) calls in public debate to enact such measures. Combined with their significant economic implications for the country, as noted, it might be expected that they were deemed premature, excessive, or otherwise controversial. Yet even without the depiction of existential threat—without the securitization of COVID-19 as supposed in the original framework—the extraordinary measures enacted by the Ardern administration were widely supported by the population. One poll, conducted *during* the lockdown, suggested that 87 percent of New Zealanders approved of the way the government was handling the virus (in Hollingsworth 2020).

New Zealand's measures were also broadly successful (assessed in terms of cases and fatalities) and supported beyond the period addressed here. By the end of 2020, New Zealand still had fewer than 7,000 cases nationally, had experienced only twenty-five fatalities, and had not needed to return to national restrictions put in place in April. While its borders remained closed to international arrivals, the country clearly avoided a devastating second or third wave of the virus evident in so many other states, including the United Kingdom. And Jacinda Ardern was reelected comfortably in October 2020, in an election many interpreted as an endorsement of the government's handling of the pandemic.

## Conclusion

A range of states—beyond those discussed here—used different forms of justification for similar sets of extraordinary measures to address the perceived challenge posed by the coronavirus. In some cases, the direct linkage between representations of threat, of existential security, of the unavoidability of extreme responses appears to align neatly with the expectations of securitization theory. In countries such as France, for example, representations of extraordinary threat were tied to the imperative of exceptional responses, with French President Macron declaring “we are at war” while announcing unprecedented national restrictions (DW 2020). And in some instances, the apparent use of the cover of the coronavirus to expand or consolidate government powers (e.g., Hungary) or the surveillance regime of the state (e.g., China) appears to further validate the concerns of some scholars that—as a general rule—it is preferable to avoid securitization (see Caso 2020; Musu 2020; Sondermann and Ulbert 2020).

Yet the analysis undertaken here suggests significant degrees of variation in terms of the ways in which emergency measures were justified and enabled in the context of COVID-19, and the associated significance of representations of threat to this process. Two empirical findings are particularly pertinent here.

First, despite being the concern of many commentators, representations of threat or the “war metaphor” were not always the decisive representation. In New Zealand, as noted, it simply was not the case that securitizing moves were especially prominent—the Prime Minister seemed to studiously avoid representations of threat, war metaphors, or invocations of existential security in outlining or justifying exceptional measures. And even when these representations were used, they were not necessarily securitizing in the traditional sense: Australia merged comparisons to World War II with representations of potential and future harm that are more in line with risk and paired its emphasis on centralized executive power with appeals to solidarity and citizen responsibility.

Second, expectations around extreme (decisionist) measures in response to COVID-19 requiring equally extreme language do not seem to hold up, especially when context is added. While the UK leadership was experiencing significant domestic pressure to pursue serious measures aimed at arresting the spread of the virus by the time these measures were ultimately announced and pursued, New Zealand's extraordinary measures were pursued at a point at which the country had experienced very few cases and was under minimal pressure to escalate the national response. In this sense, it would be expected that the United Kingdom’s leadership would have less to do to justify extraordinary measures than their New Zealand counterparts, suggesting that more alarmist rhetoric might be expected from New Zealand than the United Kingdom. Yet the opposite was true, at least in terms of representations of existential threat.

These findings serve to challenge dominant accounts of COVID-19 and its “securitization” in important ways, but the analysis here also has implications for the securitization framework more broadly.

First, these findings illustrate that dominant conceptions of securitization as an elite-driven process, in which leaders point to an existential threat to justify exceptional responses, are not necessarily borne out in practice. The example of the United Kingdom could feasibly be interpreted as an example of ground-up securitization, in which a range of actors at a level below the political leadership were conceiving and conceptualizing the coronavirus as an existential threat long before its leaders embraced pronouncements linking the COVID-19 fight to security and the experience of war. Alternatively, we might conclude that the UK example points less to how political leaders enable exceptional measures through securitization and more to how security “language” serves to signal a level of concern with an issue consistent with societal expectations. Either way, the model of a political elite employing the language of security and threat in order to justify exceptional measures that might not otherwise have been countenanced—evident in both the theory's development and its application (see Wæver 2011, 469)—fits uneasily with this example. This is particularly significant because, at a superficial level, the UK example appears as a textbook case of securitization given the use of security language by elites and the deployment of exceptional measures.

Second, these findings suggest that exceptional measures—in which polities break from normal procedure and practices to pursue extreme responses—should not automatically be assumed as the product of securitization. As noted earlier, this assumption derives from the core framework's use of Schmitt and is explicitly evident in Buzan and Wæver's *Regions and Powers* (2003, 73). In that book, the pursuit of extraordinary measures is taken as evidence that securitization has occurred. The New Zealand case here suggests that this is a problematic assumption. To reiterate, while extraordinary measures were clearly pursued—in the form of border closures and national lockdowns—the preceding depiction of coronavirus as a security threat was almost wholly absent from the Prime Minister's announcement of these measures, despite the timing arguably making alarmist language more necessary than in the other cases discussed. Rather than the threat of immediate and violent death assumed by the framework, Prime Minister Ardern justified emergency measures through emphasizing the risk of COVID-19’s spread, the need to manage this possibility, and the imperative of (national) solidarity. Ultimately, this analysis supports recent accounts of the different ways that exceptionalism can be sanctioned and enacted, including via the language of risk (see Kirk 2020).

None of the above fundamentally questions the broader utility or contribution of the securitization framework. In many ways, it should serve to reaffirm and echo broader calls for context-oriented examinations of the effects of security speak and the processes through which emergency measures become possible (see McDonald 2008; Stritzel 2007; Ciuta 2009; Balzacq 2019). In the process, the preceding analysis should serve to raise important questions about the politics of exceptionalism. Do securitizing moves and securitization do what we think they do? Do such moves serve to enable emergency measures or can they be encouraged or even compelled by societal expectations? And how are emergency measures themselves rendered feasible and possible in different social and political contexts? While tempting to view the use of narratives of security, threat, and war in response to COVID-19 as a textbook case of securitization—its effects and the dangers associated with it—this comparative analysis points to the importance of more nuanced and contextual analysis that examines the different ways in which such questions play out in different contexts. As such, it encourages us to examine—rather than assume—the politics of security.

## Acknowledgments

The authors would like to thank the editors and the reviewers for their thoughtful comments on this paper, and Al Stark and Allan McConnell for feedback on an earlier iteration of this paper.

## Footnotes

1 An exception here is Buzan and Waever's *Regions and Powers* (2003, 73), in which the authors focus on “broad indicators of securitization rather than investigating each instance in detail,” and “emergency measures (are taken) as indicators of securitization.”

2 In the case of the United Kingdom, this is augmented by analysis of a speech by Health Secretary Matt Hancock.

3 As of January 12, 2021, Australia has reported 28,634 cases, with 909 deaths.

4 Stage 4 lockdowns in New Zealand involved the closure of all nonessential businesses, suspension of all unnecessary travel, limits on contact with others and the closure of all schools, universities, and childcare services (see Fuller 2020).
